# Factors Influencing the Survival of Preformed Zirconia Crowns in Children Treated under General Anesthesia

**DOI:** 10.1155/2021/5515383

**Published:** 2021-03-17

**Authors:** Abdurahman S. Alhissan, Sharat Chandra Pani

**Affiliations:** ^1^Ministry of Health, Riyadh Kingdom of, Saudi Arabia; ^2^Schulich School of Medicine and Dentistry, University of Western Ontario, London, ON, Canada

## Abstract

**Aim:**

This study aimed to retrospectively evaluate the success of zirconia crowns placed in the anterior teeth of children and evaluate the impact of pulp therapy of the tooth on the rate of failure.

**Materials and Methods:**

A total of 70 anterior teeth of 20 children aged between 3 and 5 years who had undergone the placement of zirconia crowns under general anesthesia were followed up for 24 months. Kaplan–Meier Survival curves were plotted for the estimation of two-year survival time. The outcomes for teeth that had received pulp therapy were compared to those that had not received pulp therapy.

**Results:**

Kaplan–Meier survival analysis of 70 crowns observed over a two-year period showed that mean survival time for the crowns was 38.7 months with a confidence interval ranging from 38.1 months to 39.3 months. When the survival of the crowns was observed based on the presence or absence of symptoms, it was observed that only 4 out of the 70 crowns were symptomatic (with or without crown loss) at the end of two years, giving a success rate of 94.3%. The mean survival time was also increased to 39.5 months (confidence interval 39.15–39.98 months).

**Conclusion:**

Zirconia crowns provide an acceptable level of success and longevity. Crowns placed on teeth after pulp therapy are more likely to fail than those placed on teeth without pulp therapy.

## 1. Introduction

Dental treatment under general anesthesia is often the only means to manage very young children with extensive dental caries. It has been estimated that up to 80% of children who are treated under general anesthesia are below 4 years of age [1]. In the past, the restoration of badly destructed primary incisors relied on the placement of resin composite “strip crowns” and the use of veneered stainless steel crowns (SSCs) [2, 3]. Studies have shown that failure rates for composite resin restorations placed under general anesthesia are between 29% and 45% [1, 4]. Traditionally, the restorative treatment of children under general anesthesia has focused on aggressive treatment of the dental caries, with full coverage SSCs being the treatment of choice [1, 4, 5].

There has been an increased demand for aesthetic anterior restorations in children with both parents and children showing increased need for aesthetics [3, 6]. Over the past decade, the introduction of preformed zirconia crowns has radically altered the way pediatric dentists view aesthetic dentistry [3, 7–10]. There have been several studies documenting the use of zirconia crowns in badly destructed primary teeth [9, 10]. While there have been a few studies documenting success rates of these crowns, data from retrospective cohort studies are still sparse [9, 11].

The role of oral hygiene and systemic causes of periodontal disease on the overall oral health of individuals is an important one [12, 13]. Despite the low incidence of periodontal disease in children, there is a history of poor oral hygiene in Saudi Arabia. Studies have shown that the rates of dental caries in children below five years of age are greater than 85% [14]. There is also literature showing that there is a high rate of secondary caries and failure of restorations placed in children in Saudi Arabia [15, 16]. However, there is a lack of data on the causes of failure of zirconia crowns in Saudi Arabia. This study aimed to retrospectively evaluate the success of zirconia crowns placed in the anterior teeth of children and evaluate the impact of pulp therapy of the tooth on the rate of failure.

The impact of pulp therapy was assessed using the null hypothesis that there is no significant difference in the failure rates of zirconia crowns placed on teeth with pulp therapy and crowns placed on teeth without pulp therapy.

## 2. Materials and Methods

The study followed a retrospective cohort study design. Ethical approval for the study was obtained from the Institutional Review Board of the Riyadh Elm University (FPGRP/43735005/236).

### 2.1. Sample Selection

**Figure 1:**
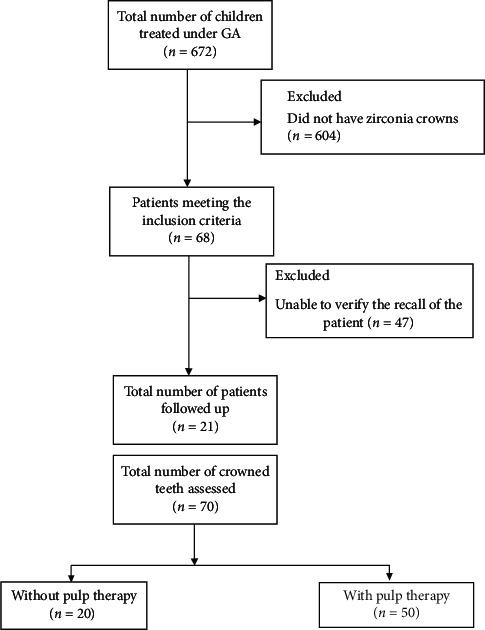
Study design.

The files of all pediatric patients treated under general anesthesia between January 2012 and January 2018 in Riyadh Elm University, Riyadh, Saudi Arabia, were screened for patients below 71 months of age who had had one or more zirconia crowns (NuSmile®, NuSmile Dental Florida, USA). The files of children who met the inclusion criteria were screened to extract information regarding follow-up visits. For patients who had completed six-month recall visits for a period of two years (or failure of the crown, whichever was earlier), data regarding the success and failure of the restoration were recorded (Figure 1).

### 2.2. Recorded Data

The status of the restoration was recorded using dichotomous success-fail criteria, whereby a crown was said to have failed if one of the following points was observed:

- Uncomplicated debonding of crown from the tooth—teeth where the crown was debonded from the prepared tooth surface; however, the patient did not suffer from pain or abscess.
- Complicated debonding of the crown—teeth where the crown was debonded and the patient had suffered from a complication, such as pain, abscess, or mobility of tooth
- Complications with crown intact—where the crown was not affected but the patient complained of pain or food impaction and/or showed clinical signs such as abscess or tooth mobility
- Integrity of the crown was compromised with or without secondary caries

Success of the crowns was recorded at the end of the two-year follow-up period (24 months), while the failure of the restoration or the presence of secondary caries was recorded at the follow-up interval at which it was first noticed (3, 6, 12, 18, or 24 months). For patients who reported back with a failed restoration, the time elapsed since the placement of the restoration was recorded.

### 2.3. Statistical Analyses

Descriptive statistics were tabulated keeping in mind the different failure criteria described. Statistics on the age of the patient, number of crowns placed, and overall survival rate were also tabulated. The differences in parametric variables such as age were compared using the independent *t*-test, while differences in nonparametric variables were compared using the chi-square test.

The failure of the crowns was extrapolated into separate Kaplan–Meier survival curves for teeth with and without pulp therapy, along with an overall survival curve. Similar curves were used to compare the type of failure. ROC curves were plotted to correlate between failure of the crown and the presence or absence of pulp therapy. All statistics were computed using the IBM-SPSS ver. 25 data processing software (IBM Corp., Armonk NY, USA).

## 3. Results

The sample consisted of 20 patients (9 males and 11 females) aged between 31 and 40 months at the time of placement of the crown (mean age = 32.14 months, SD ± 2.1 months). All patients were diagnosed with severe early childhood caries, and the treatment of all children had been performed under general anesthesia. There was no significant difference in age between the boys (31.12 months, SD ± 3.2 months) and the girls (34.12 months, SD ± 1.3 months) in this study (*p*=0.356).

Of the 70 teeth studied, a significant majority had undergone pulp therapy (*n* = 50). Among those, most had undergone pulpectomy with only a small minority (*n* = 6) having undergone pulpotomy. Debonding was the major cause of failure in both groups, with pulpal complications (with or without debonding) being observed only in the pulpally treated group (Table 1).

**Figure 2:**
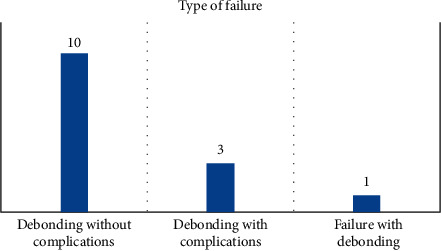
Types of failure observed.

The overall success rate of the zirconia crowns was tabulated using the Kaplan–Meier survival analysis. Of the 70 crowns observed over a two-year period, 14 crowns were classified as clinical failures, giving a success rate of 80%. However, most of those failures were bond failure (*n* = 10) (Figure 2). The mean survival time for the crowns was 38.7 months with a confidence interval ranging from 38.1 months to 39.3 months.

**Figure 3:**
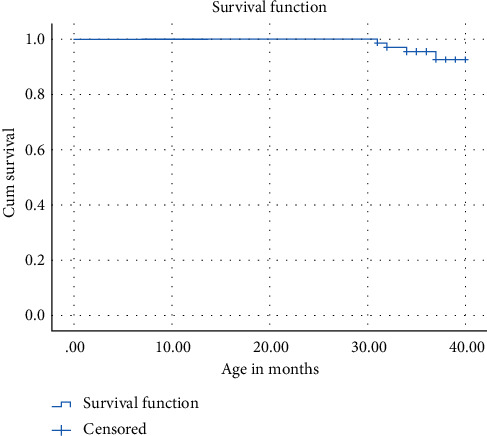
Kaplan–Meier survival curve for mean survival time of crowns.

The success or failure of each crown was then tabulated in a Kaplan–Meier table to compute a Kaplan–Meier curve to show the overall survival pattern of the zirconia crowns (Figure 3). When the survival of the crowns was observed based on the presence or absence of symptoms, it was observed that only 4 out of the 70 crowns were symptomatic (with or without crown loss) at the end of two years, giving a success rate of 94.3%. The mean survival time was also increased to 39.5 months (confidence interval 39.15–39.98 months).

**Figure 4:**
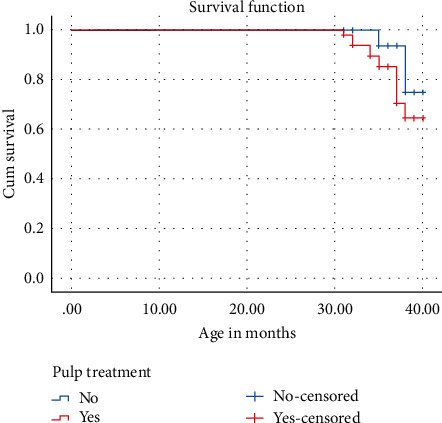
Kaplan–Meier curve showing the comparison between teeth with and without pulp therapy.

The success rate of the crowns with pulp therapy (76%) was significantly lower than the success rate of the crowns without pulp therapy (90%). The chi-square test found this rate to be statistically significant (chi-square = 48.12, *p* < 0.001). The mean survival time of the teeth with pulp therapy (39.32 months, SD ± 1.4 months) was lower than the mean survival time of teeth that were not pulpally treated (38.45 months, SD ± 1.3 months). The survival functions for the crowns with pulp therapy and those without pulp therapy were plotted using Kaplan–Meier survival charts (Figure 4).

These results suggest that the null hypothesis be rejected and that teeth with pulp therapy have a significantly worse outcome after restoration with zirconia crowns than teeth without pulp therapy.

## 4. Discussion

The advent of preformed zirconia crowns has changed the level of parental aesthetic expectations in pediatric dentistry [8–10, 17, 18]. The introduction of these aesthetic and relatively easy to use crowns has meant that parents can now demand and receive high-quality anterior aesthetic full coverage restorations in primary anterior teeth [18]. Despite the higher cost of these crowns, zirconia crowns remain affordable, and there has been a marked increase in their use worldwide [19].

The use of general anesthesia for dental treatment has been a controversial topic. While the extent of caries and the complexity of care often necessitate care under general anesthesia, the use of the modality also significantly increases the cost of dental care [20–22]. In the current study, the rationale for evaluating only crowns placed under general anesthesia was based on the rationale that the use of multiple crowns in very young children is an indicator of early childhood caries.

While at the outset, it may seem necessary to pulpally treat teeth indicated for zirconia crowns, and there is no definitive rationale for the use of pulp therapy with zirconia crowns in teeth where the dental caries do not reach the pulp [23]. Clinical examination is often the only means to accurately identify the pulp status of primary teeth [23]. In this regard, the decision to perform pulp therapy or not was based on whether clinical pulp exposure was observed or not.

Our results show that pulp therapy did not significantly alter the outcome of the crowns, with teeth that were pulpally treated showing higher failure rates. One of the reasons for this could be the fact that those teeth that needed pulp therapy had less tooth structure than the teeth that were not pulpally treated. This is significant given that the greatest type of failure observed was debonding of the crown.

It is a documented fact that despite the care given to crown preparation, the cement is the principal source of retention for zirconia crowns. In this regard, it is not surprising that most of the failure rates reported were for debonded crowns. Even with this relatively benign failure, the projected success rate for zirconia crowns in this study (80%) was higher than reported rates for composite resin-based strip crowns [1, 5, 24]. When debonding is ignored, the success rates are comparable if not greater (96%) to those reported for preveneered SSCs or open-faced SSCs [25, 26].

The results of this study must be viewed keeping in mind certain limitations. This study was a retrospective study, and in that regard, the results are not as powerful as those from the split mouth clinical trial. However, Kaplan–Meier survival curves have been shown to be a valid tool for the prediction of success of restorations placed in children [27]. The role of oral hygiene and the impact of oral hygiene on the overall survival of the crown is a factor that was beyond the scope of the current study. Furthermore, the limited number of individuals in the study do not allow for the control of factors such as socioeconomic status of the parents or oral hygiene and oral hygiene practices. Despite these limitations, this study provides an insight into the clinical potential of these crowns.

## 5. Conclusion

Within the limitations of the current study, we can conclude that zirconia crowns provide an acceptable level of success and longevity. Crowns placed on teeth after pulp therapy are more likely to fail than those placed on teeth without pulp therapy.

## Figures and Tables

**Table 1:** Overview of the type of treatment and the failures observed in each group.

		Pulp treatment		Total
		No	Yes	
Failure	No clinical failure	18	38	56
	Debonding without complications	2	8	10
	Debonding with complications	0	3	3
	Failure without debonding	0	1	1

Total		20	50	70

## Data Availability

The data used to support the findings of this study are available from the corresponding author upon request.
